# Findings from a prospective cohort study evaluating the effects of International Health Advisors’ work on recently settled migrants’ health

**DOI:** 10.1186/s12889-017-4273-0

**Published:** 2017-04-28

**Authors:** Susanne Sundell Lecerof, Martin Stafström, Maria Emmelin, Ragnar Westerling, Per-Olof Östergen

**Affiliations:** 1 Social Medicine and Global Health, Department of Clinical Sciences Malmoe, Lund University, Clinical Research Centre, Jan Waldenstroms gata 35, 205 02 Malmoe, Sweden; 2 Department of Public Health and Caring Sciences, Uppsala University, Box 564, 751 22 Uppsala, Sweden

**Keywords:** migrants, health promotion, health communication, health information, social determinants of health, social capital

## Abstract

**Background:**

Several interventions have been carried out to tackle health inequalities between migrant groups, especially refugees, and native-born European populations. These initiatives are often address language or cultural barriers. One of them is the International Health Advisors (IHA) in Sweden; a peer education intervention aimed at providing health information for recently settled migrants. It is known that social determinants, such as educational level and access to social capital, affect health. Social determinants may also affect how health information is received and transformed into practice. The aims of this study was to a) assess the impact of the IHA on recently settled migrants’ self-reported health status, and received health information; b) determine the moderating role of educational level and social capital; and c) critically discuss the outcomes and suggest implications for health promotion practice.

**Methods:**

The study was designed as a prospective cohort study. A postal questionnaire translated to Arabic was sent to recently settled Iraqi migrants in eight counties in Sweden, in May 2008 and May 2010. Two of the counties were exposed to the intervention, and six were used as references.

**Results:**

The proportion of individuals who reported that they had received information on healthy diet and physical exercise was higher in the intervention group than in the non-intervention group (OR 2.31, 95% CI 1.02–5.22), after adjustments. Low social participation was negatively associated with deteriorated or unchanged health needs (OR 0.47, 95% CI 0.24–0.92). No other statistically significant differences in health outcomes could be observed between the groups. No signs of effect modification on this association by social capital or educational level could be found.

**Conclusions:**

Health information provided by the IHA increased self-reported level of knowledge on healthy diet and physical exercise. The interpretation of the observed negative association between low social participation and deteriorated or unchanged health needs is that participation was limited to one’s own social group, and therefore had limited positive influence on health seeking behaviour. The lack of measurable improvements in health status could be explained by limitations in the study, in the theoretical assumptions underlying the intervention, and in the implementation of the intervention. Further research is needed to understand success factors in health promoting interventions among recently settled migrants better.

## Background

Migrant groups, especially refugees, have poorer health status than native populations in for example Europe and the US. Their self-rated health has been found lower [1–3], their mental health poorer [4–10], and they have a higher proportion of unmet health needs [11]. Previous studies also show lower health literacy rates and lower access to health information [12–15]. However, some studies have indicated that these associations can be moderated by educational level [12]. These circumstances threaten health equity irrespective of ethnic background or socioeconomic status.

The described situation poses a challenge to health promotion practice. Barriers to provision of adequate health services and health promotion interventions have been reported in the literature, e.g. language problems, cultural differences and lack of trust [16–19]. The term ‘migrant’ is defined by the International Organization of Migration as: “Any person who is moving or has moved across an international border or within a State away from his/her habitual place of residence, regardless of (1) the person’s legal status; (2) whether the movement is voluntary or involuntary; (3) what the causes for the movement are; or (4) what the length of the stay is”, and the term ‘refugee’ is defined by the United Nations Refugee Agency as “Any person who, owing to well-funded fear of being persecuted for reasons of race, religion, nationality, membership of a particular social group or political opinion, is outside the country of his nationality and is unable or, owing to such fear, is unwilling to avail himself of the protection of that country; or who, not having a nationality and being outside the country of his former habitual residence as a result of such events, is unable or, owing to such fear, is unwilling to return to it”. Hence, recently settled migrants are a heterogenous group with different socioeconomic backgrounds and differential access to social capital in their daily environment. Given different migration-related factors, together with social determinants, may play important roles for the health development in the new country and for how health interventions are received and transformed into action.

Different strategies have been adopted to address health inequities. One of the most common strategies utilized is to provide health information to specific target groups. Health information is believed to increase knowledge that in turn will help people to make rational and informed health choices. However, this approach has been criticized for making simplistic assumptions about how health is created. Social cognitive theory was developed by the Canadian psychologist Albert Bandura in 1986. Bandura [20] claims that such a strategy does not take into account the social and economic factors that may play a mediating role or affect people’s health directly, for example educational level and social capital. Persons with higher educational level seem to find it easier to interpret and adopt information, and to translate it into practice [21]. People with high access social capital, measured as social participation and trust in others [22], have also been shown to have better health outcomes [23]. Social capital is believed to affect health through social interaction or by facilitating stress-buffering [24]. Social interaction can promote positive or negative health-related behaviors through shared norms, whereby belonging to a social network can help individuals access both tangible and intangible resources in times of need. The stress-buffering model suggests that individual’s perceived access to practical and/or emotional social support alleviates discomfort under stressful life events, and thereby moderates the individual’s emotional and behavioral response to that event (ibid).

In Sweden, several health-promoting interventions have been conducted among recently settled refugees in order to promote health and/or prevent ill health. In this study, we examine a peer-to-peer health information intervention known in Sweden as “International Health Advisors” (henceforth IHA). The IHA are persons with migrant background, who are employed by the county board or county council, and offer their recently settled peers health and health services information in their native language. Some interventions, also targeting migrant groups, have been scientifically evaluated. Ekblad and colleagues [25] found that a group of recently settled Arabic- and Somali-speaking women who received a culturally tailored health promotion intervention carried out by licensed clinicians improved their self-rated health and self-reported level of strength and energy significantly during the intervention. Qualitative interviews indicated that the women felt they had better knowledge and preparedness to manage stress (ibid). Another culturally adapted intervention aimed at reducing diabetes risk through dietary advice, was implemented among Pakistani women in Oslo, Norway. Norwegian project staff did the teaching and led the discussions with the help of interpreters. The study showed that the women improved their knowledge on healthy diet, and increased their intentions to change their dietary habits in order to reduce weight and risk of diabetes [26–28]. However, few scientific evaluations have explored the possible links between health outcomes and social determinants such as educational level and social capital and used a critical public health perspective to discuss the results with the purpose of developing a stronger evidence base.

The overall aim of this study was to assess the impact of an IHA intervention among recently settled Iraqi migrants in Sweden, by determining the change in self-reported health measures in two cohorts over a 2-year period. Contributing to better health among recently settled refugees was one of the goals of the intervention. The specific objectives were therefore to assess the impact of the intervention on self-reported health status, unmet health needs, received health information, and the role of educational level and social capital (defined as social participation or trust in others) in this; and to critically discuss the outcomes and suggest implications for development of practice.

## Methods

### Study design

This was a prospective cohort study following Iraqi citizens in eight counties in Sweden of which two received the IHA intervention and the remaining six counties were used as references.

### The intervention and its context

In this study we examine a peer-to-peer health information intervention known in Sweden as “International Health Advisors” (IHA). As mentioned before, the IHA are persons with migrant background, who are recruited and employed by the county administrative board or county council, qualified through language skills, communication skills and sometimes with a professional background in health or social care. They receive preparatory training for their special task to provide recently settled refugees with health and health services information in their native language. Further in-service training includes short courses in for example first aid. The themes covered in their information package for recently settled refugees include information about the Swedish health care system, self-care, healthy diet, physical exercise, common conditions like flu or fever and mental health. At the time of data collection, refugees usually met the IHA 1–3 times.

Recently settled refugees in Sweden receive governmental reimbursement during the first 2 years upon resettlement. The labor office is responsible for coordinating activities for the refugees and for those family members who arrive within 2 years after resettlement of the first refugee. All are offered Swedish For Immigrants (SFI), civic information, and labor market introduction, unless they are on parental leave or sick leave. The intervention took place mainly in Swedish for immigrants’ classes for targeted groups of recently settled refugees. In the two counties that had access to IHA during the time of data collection, health information was also part of these activities offered for the recently settled refugees. At the time of data collection, the Iraqi migrants were the largest migrant group coming to Sweden, the majority of them as refugees or family reunion cases of refugees, ensuring a sample size large enough. The rationale was to minimize as much as possible the effects of different cultural backgrounds, different reasons for and different time periods of migrating.

### Material

Data was collected through a postal questionnaire. The questionnaire was designed in a group of researchers (including the authors) in a project aimed at evaluating and contributing to the improvement the intervention. The community was consulted in a qualitative pilot study.

The questionnaire was translated to Arabic and tested for comprehensibility and cultural appropriateness in focus groups stratified for gender and educational level. After revisions, it was sent out to all adults born in Iraq who were identified as new individuals in the Swedish population register in the counties of Stockholm, Uppsala, Södermanland, Östergötland, Örebro, Västra Götaland, Kronoberg and Skåne during the time period December 1st 2007 – February 28th 2008. At this time, the intervention of IHA was established in the counties of Skåne and Östergötland. The exposed and unexposed counties were similar in degree of urbanization, employment an immigration rates, and the system for receiving recently settled refugees is the same in all counties in Sweden. To our knowledge, there were no systematic factors affecting recently settled refugees’ choice of county for resettlement.

The baseline questionnaire was administered in May 2008 and resulted in a response rate of 51% (*n* = 617/1213). Twenty-nine per cent of the respondents (*n* = 181/617) resided in the intervention counties and 71% (*n* = 436/617) in the non-intervention counties. 68% (*n* = 421) of the persons who answered the baseline questionnaire also answered a follow-up questionnaire in May 2010. Twenty-one per cent (*n* = 117) of them resided in the intervention counties and 79% (*n* = 286) in the non-intervention counties.

The study was approved by the Regional board of ethical vetting in Lund (reference numbers 2009/22 and 191/2008),

### Variable definitions

The intervention variable was defined by county of residence at baseline and follow-up, assuming that the study subjects who remained residents in the counties of Skåne and Östergötland would, at some point, be exposed to contact with the International Health, and that those residing in the other counties would remain unexposed to the intervention. Those who moved from an intervention county to a reference county, or vice versa, during the study period were omitted from the analyses (*n* = 18). The reason for choosing this intention-to-treat approach was to attempt making a “real world” evaluation of the intervention, namely to estimate what would most likely be the effect if the interventions was repeated in a new setting, rather than to evaluate the effect only among those selected individuals who were exposed to the intervention activities at a certain level (usually a level which would be likely to have an effect). This means a conservative approach, but we argue that the yielded information is more valuable for those contemplating to implement a similar intervention program.

The outcome variables were *unmet health care needs, poor mental health, poor dental health status, poor self-rated health, long-term illness, social capital measured as social participation and trust in others,* and *received information on diet, physical exercise and dental health.* The outcome variables were coded focusing on change, not absolute levels. “Negative change”, “no change” and “positive change” represent three values on an ordinal scale, and the variables were dichotomized between “positive change” and the other two variable values on this scale.

*Unmet health care needs* were measured by asking the question: “Have you ever, during the last 3 months, regarded yourself in need of medical care, but not sought a doctor?”. The variable was dichotomized, so that the response alternative “yes, several times”, indicating that health needs persisted, was defined as having unmet health care needs, and “yes, once” and “no” were defined as not having unmet health care needs. The values from the dichotomized baseline variable were combined with the dichotomized follow-up variable, creating a single variable with four values signifying change between baseline status and follow-up: “Remained good”, “improved”, “remained bad” and “deteriorated”. The new variable was then dichotomized into two values coded as “Improved” and the other three values as “unchanged or deteriorated”.

*Mental health* was assessed by using the 12-item instrument General Health Questionnaire, GHQ-12, developed by Goldberg and colleagues [29]. The instrument has been translated to several languages, including Arabic [30, 31], and has been found to have a good cross-cultural validity [32, 33]. The 12 items in the instrument were dichotomized so that values indicating symptoms of poor mental health “as usual” or “more than usual” was coded as 1, and “less than usual” or “a lot less than usual” was coded as 0. The dichotomized variables were then added to each other so that a scale rendering 0–12 points was created. The mean of the distribution of the points in the sample was 2.65, and the cutoff point was set between two and three when the point scale was dichotomized. This cutoff was similar to that applied by other studies [32]. *Poor mental health* was defined as having three points or more on the scale. The baseline and follow-up variables were combined in a new variable signifying change between baseline and follow-up, which was coded with the values “improved”, “remained good”, “remained bad” and “deteriorated”. Finally, the combined variable was dichotomized in the same way as the previous change variable.

*Dental health* was measured by asking the respondents to rate their dental health on a Likert scale with five values; “Very good”, “Quite good”, “Neither good nor poor”, “Quite poor” and “Very poor”. The baseline and the follow-up variables were dichotomized, so that those who rated their dental health as “quite poor” or “very poor” were defined as having *poor dental health,* and the others as having *good dental health.* A change variable was constructed, coded and dichotomized in the same way as for the previous outcomes.

Similarly, *self-rated health* was assessed with a single question: “How would you currently assess your general health?” – requiring the respondents to rate their current general health status on a Likert scale with five values; “Very good”, “Quite good”, “Neither good nor poor”, “Quite poor” and “Very poor”. The baseline and follow-up variables were dichotomized so that “Quite poor” or “very poor” were defined as *poor self-rated health,* and the other values as *good self-rated health.* This instrument has demonstrated good predictive value when it comes to actual morbidity and mortality [34, 35]. The change variable was then developed in the same way as the previous outcome variables.

*Long-term illness* was defined as having answered “yes” to the question “Do you have any lingering illness, difficulties following an accident, disability or other frailty. The baseline and follow-up variables were transformed into a change variable similarly as the previous outcome variables.

*Social participation*, a component of social capital, was assessed using the question “How often do you attend a meeting or another activity in any organization or group (for example sports organization, interest organization, mosque or church, women’s or men’s group)? The scale was used in the Malmo Diet and Cancer Study [36] Both the baseline and the follow-up variable was dichotomized, so that reporting less often than monthly was defined as *low social participation*, and the other values as having *high social participation*, indicating social participation on a regular basis*.* The dichotomized baseline and follow-up variables were combined, constituting a change variable that was also dichotomized in the same way as the other outcome variables.

*Trust in others*, another component of social capital, was assessed by combining four variables which measured horizontal trust: “Take a stand on the following statements: a) Most people would use you if they got the chance b) Most people essentially try to be fair c) You can trust most people and d) You cannot be careful enough when you deal with other people”. The response alternatives were “Do not agree at all (0 points)”, “Do not agree (1 point)”, “Agree (2 points)”, “Completely agree (3 points)”. The second and the third variable were recoded in order to make the scales go in the same direction, with higher values indicating lower trust. The combined scale was dichotomized at the 75th percentile of the distribution of points in the sample. Seven points or more on the scale was defined as *low trust in others.* A change variable, made up from the baseline and the follow-up values, was constructed and then dichotomized in the same way as all the previously described ones.

The three variables *received information on healthy diet, received information on physical exercise* and *received information on dental health* were measured by asking the respondents “Have you received information on healthy diet/physical exercise/dental health?” (three different questions) and the response alternatives were “Yes, a lot”, “Yes, quite much”, “Yes, a little” and “No”. For the regression analyses, we constructed a combined change variable by combining the baseline and follow-up variables information on healthy diet and information on physical exercise, so that the responses created an index of 16 different combinations of the values. They were combined using the same logic as when constructing a measurement (“scale”) consisting of more than one item, which measures similar, but not identical, aspects of an underlying concept such as work stress or alcohol addiction. This resulted in a better precision of the underlying concept which could be labelled “health education on healthy lifestyles related to increased risk for overweight/obesity”. We then dichotomized this new variable into a response category, which corresponded to having received information on at least one of the topics healthy diet and physical exercise during the study period, and a group which was made up by all other persons in the sample. Similarly, the change variable for baseline and follow-up information on dental health was dichotomized into a response category which meant that the individual had received information during the study period, and a group consisting of all other individuals in the sample.

The variables we adjusted for in analyses were *age, gender* and *educational level*. The variables *gender* and *age* were derived from the Swedish population register. *Educational level* was measured by asking the question “What education do you have”, with the response alternatives “none”, “1–6 years”, “7–9 years”, “10–12 years”, “academic education (university, college)” and “other education, what?”. “Other education” was a mixed category of which a majority consisted of persons with higher academic degrees or vocational training, and was therefore recoded to “High level of education”, together with those who reported at least 10 years of education. Seven to nine years of education or less was defined as *low educational level.*

### Statistical methods

Data were analyzed with the statistics software package IBM SPSS Statistics 22®. We calculated frequencies and percentages of basic sociodemographic characteristics of the sample, for all, and stratified by intervention and non-intervention counties. We also calculated the *p*-value of within-group differences for health outcomes, health information and determinants, between baseline and follow-up using McNemar’s test, set at the 95% significance level. We then performed logistic regression analyses to calculate crude odds ratios and 95% confidence intervals for health outcomes and health information by the different determinants. We also analyzed the between-group differences for the variables that were statistically significant in the binary analyses, using multivariate logistic regression. Finally, we performed analyses of effect modification by social capital (social participation and trust in others) on the associations that were statistically significant in the binary analyses.

## Results

Our sample consisted of 403 persons, of which 167 (41.4%) were men (Table 1). The distribution of the genders was very similar in the intervention (*n* = 117) and the non-intervention group (*n* = 286). Mean age was 37.2 in the intervention group and 38.1 in the non-intervention group. The intervention group had a larger proportion of persons in the age group 31–45 (48.7%) than the non-intervention group (45.1), whereas the non-intervention group had a larger share of persons aged 46 and over (27.4%) than the intervention group (18.8%). Low educational level was more common in the intervention group (35.7%) than in the non-intervention group (27.4%).

**Table 1:** Comparison of the distribution of sociodemographic variables, health outcomes and health information at baseline between intervention and non-intervention area

Variable	Total (*N* = 403)	Intervention area (*N* = 117)	Non-intervention area (*N* = 286)	*p*-value^1^
Male	41.4% (167)	41% (48)	41.6% (119)	0.91
Female	58.6% (236)	59% (69)	58.4% (167)	
Mean age	37.8	37.2	38.1	
Participating in SFI^a^	70.1% (410)	74.5% (82)	68.3% (276)	
Low educational level	29.8% (116)	35.7% (40)	27.4% (76)	0.11
Low social participation	74.4% (291)	73.5% (86)	71.7% (205)	0.77
Low trust in others	41.6% (157)	36.7% (40)	43.7% (117)	0.21
Unmet health needs	41% (129)	47.8% (44)	38.1% (85)	0.11
Unmet dental health needs	47.2% (149)	56% (51)	43.6% (98)	0.04
Poor mental health	30.9% (121)	31.6% (36)	30.7% (85)	0.86
Poor self-rated health	16.6% (62)	19.6% (21)	15.4% (41)	0.32
Long-term illness	26.9% (105)	26.5% (30)	27% (75)	0.93
No information on healthy diet	40.5 (160)	45.2% (52)	38.6% (108)	0.22
No information on physical exercise	65.7% (261)	70.7% (82)	63.7% (179)	0.18
No information on dental health	63.8% (254)	70.7% (82)	61% (172)	0.07

^a^ Swedish for foreigners

^1^ *P*-values were obtained by performing McNemar’s test

The proportion of respondents who reported unmet health needs and unmet dental health needs had decreased between baseline and follow-up in both the intervention group and the non-intervention group (Table 2). However, the decrease was not statistically significant, but for the unmet health needs in the non-intervention group it just fell short of this (*p* = 0.06). When the change between baseline and follow-up was assessed for all respondents, without stratification by intervention area, it was statistically significant (*p* = 0.01). Only small, not statistically significant, changes could be seen for self-reported health status outcomes, social participation and trust in others. The proportion of respondents who reported not having received health information on health diet, physical activity and dental health decreased between baseline and follow-up. The changes in proportion of persons who reported not having received information on healthy diet (*p* = 0.02), physical exercise (*p* = 0.004) and dental health (*p* = 0.00) were all statistically significant in the intervention group. In the non-intervention group, only the change in proportion of respondents who had not received information on dental health was statistically significant (*p* < 0.001).

**Table 2:** Comparisons of change in prevalences of health outcomes and health determinants between baseline and followup, and between intervention and non-intervention area

Health outcomes	Intervention area (*N* = 117)			Non-intervention area (*N* = 286)		
	Baseline	Followup	*p*	Baseline	Followup	*p*
Unmet health needs	47.8% (44)	37.9% (36)	0.09	38.1% (85)	31.6% (72)	0.06
Unmet dental health needs	56% (51)	45.5% (45)	0.11	43.6% (98)	42.9 (94)	0.90
Poor self-rated health	19.6% (21)	19% (22)	1.00	15.4% (41)	17.5% (50)	0.43
Poor mental health	31.6% (36)	24.6% (28)	0.39	30.7% (85)	30.7% (84)	0.81
Long-term illness	26.5% (30)	30.2% (35)	0.65	27% (75)	30.6% (85)	0.51
Health determinants	Intervention			Non-intervention		
	Baseline	Followup	*p*	Baseline	Followup	*p*
Low social participation	75.4% (86)	78.4% (91)	0.86	74% (205)	71.3% (199)	0.42
Low trust in others	36.7% (40)	45.6% (52)	0.18	43.7% (117)	49.3% (133)	0.16
No information on healthy diet	45.2% (52)	32.8% (38)	0.02	38.6% (108)	36.3% (103)	0.53
No information on physical exercise	70.7% (82)	55.6% (65)	0.00	63.7% (179)	58.9% (166)	0.12
No information on dental health	70.7% (82)	48.7% (56)	0.00	61% (172)	45.7% (126)	0.00

In order to assess the change between baseline and follow-up further, we analyzed the relationships between change between baseline value and follow-up value in the outcome variables on one side and intervention status, educational level and social capital at baseline on the other (Table 3). We found statistically significant associations between not having received information on healthy diet and physical activity and living in a non-intervention area (OR 2.41, 95% CI 1.09–5.33). Low social participation was negatively associated with deteriorated or unchanged unmet health care needs (OR 0.48, 95% CI 0.25–0.92), i.e. individuals with low social participation had a lower risk to increase their unmet health care needs. These associations remained statistically significant after adjustments for gender, age, and educational level (OR 2.31, 95% CI 1.02–5.22 and OR 0.47, 95% CI 0.24–0.92, respectively, Table 4).

**Table 3:** Crude odds ratios (OR) and 95% confidence intervals (CI) for unchanged or deteriorated health outcomes and health information between intervention and non-intervention area, and regarding educational level and social capital at baseline

Outcomes:	Unmet health needs	Unmet dental health needs	Poor self-rated health	Poor mental wellbeing	Long-term illness	No info on healthy diet and physical exercise	No info on dental health
	OR (95% CI)	OR (95% CI)	OR (95% CI)	OR (95% CI)	OR (95% CI)	OR (95% CI)	OR (95% CI)
Non-intervention area	0.99	1.18	1.03	1.41	0.72	2.41	1.34
	(0.57–1.72)	(0.62–2.28)	(0.42–2.56)	(0.78–2.56)	(0.32–1.65)	(1.09–5.33)	(0.82–2.17)
Low educational level	1.0	0.86	1.0	0.76	0.77	1.38	0.79
	(0.54–2.0)	(0.5–1.46)	(0.4–2.5)	(0.41–1.42)	(0.37–1.62)	(0.54–3.54)	(0.48–1.3)
Low social participation	0.48	0.6	0.23	0.56	0.9	0.83	1.34
	(0.25–0.92)>	(0.28–1.33)	(0.05–1.02)	(0.26–1.18)	(0.39–2.06)	(0.32–2.14)	(0.81–2.23)
Low trust in others	1.07	1.65	0.54	0.62	0.99	0.8	1.12
	(0.62–1.83)	(0.83–3.27)	(0.23–1.26)	(0.35–1.1)	(0.47–2.08)	(0.35–1.8)	(0.69–1.82)

**Table 4:** Risk of not receiving information on healthy diet and physical exercise in non-intervention areas (a) and risk of not improving status of unmet health needs among persons with low social participation (b). Adjusted odds ratios (OR) and 95% confidence intervals (CI)

Independent variables	Model 1
a	
Non-intervention area	2.31 (1.02–5.22)
Female	0.98 (0.6–1.6)
Age 1.01	(0.99–1.03)
Low educational level	0.69 (0.41–1.16)
b	
Low social participation	0.47 (0.24–0.92)
Female	1.31 (0.77–2.24)
Age	1.0 (0.98–1.03)
Low educational level	0.85 (0.48–1.5)

Model 1 is adjusted for sex, age and educational level

We also analyzed possible effect modifications by social participation, social trust (social capital) and educational level on the associations between exposure to intervention and having received information on healthy diet and physical exercise, and the effect modification by educational level on the association between high social participation and unmet health care needs. None of these exposure combinations were significantly associated with the outcomes, hence there were no indications of effect modification by social participation, social trust (social capital) or educational level.

## Discussion

The evaluation of the health information intervention given to Iraqi refugees who had settled in Sweden by a special cadre of health educators with immigrant background themselves, showed that the proportion of individuals who reported unchanged or deteriorated status concerning self-rated health, mental health, or unmet health needs did not differ significantly between the intervention and non-intervention area. However, we found a statistically significant difference between the intervention and non-intervention area regarding the proportion of individuals that reported that they had received information increased between baseline and follow-up, was higher in the intervention group.

These findings suggest that the intervention of International health advisors has been successful in providing information on healthy diet and physical exercise in the intervention counties, but not in improving health outcomes. Even though the aim of the intervention was to prevent poor health, in hindsight it does not seem realistic that an intervention based only on a modest dose of information should have a significant impact on health status of the target group given the fairly short time between baseline and follow-up. Our previous research [37] has shown that alternative circumstances, like socioeconomic factors and experience of discrimination, seem to be the most important determinants for the health outcomes in the intervention group. However, the observed increase in knowledge in the intervention group regarding diet and physical activity may, if translated into practice, prevent deterioration of health in the future. This would be important, especially since previous studies have shown that immigrant groups in high-income countries are particularly vulnerable to obesity, diabetes and cardiovascular disease [38–41].

We also analyzed to what extent social capital was a factor of importance for becoming more informed about health issues, regardless of belonging to the intervention group or not. Social participation and trust are seen as important components of social capital [22]. A potential strategy for health promotion practice could be for example fostering civic engagement in neighborhoods [42]. The hypothesis, to be tested in future studies, is that targeted health information encouraging the target group to become more active in participation in society, which in the long run will build trust in others. Subsequently the target group gains better access to health services and supportive systems that is expected to affect health in a positive way. Recently settled migrants generally have weak social networks and it takes time to build up social capital, especially outside one’s own group. No positive health effects or effect modification by the social capital variables included were observed in our study. In fact high social participation had a negative effect on unmet health needs. A potential explanation for this is that recently settled migrants mainly have access to bonding social capital, through participating in networks consisting of people with similar social characteristics [43]. Bonding social capital is suggested to not only have health benefits since it actually may limit personal freedom and choice [44]. Bonding social capital may perhaps also have a negative effect on unmet health care needs if people in bonding networks consult and trust their own networks more than the institutionalized health care.

In evaluations of interventions there are commonly two types of errors. First, that the theoretical assumptions of the intervention are erroneous. Secondly, that the implementation fails to carry out the intended intervention. Regarding the first error, health inequities between migrant groups and host populations have been addressed in intervention research before, but often with a more narrow focus on a particular health behavior or outcome than that of IHA. Information is not a sufficient strategy for changing health behaviors or outcomes, as the mechanism for change includes more cognitive and environmental variables than just knowledge alteration [45]. Hence, a tailored intervention needs to be adapted to its target group in more ways than linguistically and culturally. This can be done by carrying out a thorough target group analysis (including assessment of health beliefs, health literacy and environmental factors that enable or constrain healthy choices) with a participatory approach, to guide further steps in planning an intervention. Health needs and causal mechanisms need to be established. A participatory approach would enable empowerment through opportunities to influence decision making processes, and to establish ownership of the intervention to make it sustainable. A participatory approach is also required in definition of aim and setting up feasible goals, and in choosing appropriate method and medium.

Regarding the potential implementation error, research has shown that evaluations of implementations should investigate phenomena such as fidelity, dose delivered, dose received, reach, recruitment and context [46]. The lack of measurable health effects by the IHA intervention might be explained by limitations regarding some of these criteria. First, the fidelity of the International Health Advisors’ work towards training in the programme, was challenged by the recruitment and reach of the target population. A qualitative study performed within the same research project as the one reported here, indicated that the target group did not always understand that they were part of a specific intervention [47]. This was confirmed by data in our survey, where only 42 persons (37%) in the intervention counties reported that they had met IHA. A possible interpretation of this finding is that the IHA failed to reach the whole target group, for example those who did not attend classes in Swedish for immigrants (25%, *n* = 35 in the intervention group, see Table 1). Dose delivered has been documented in the IHA s’ office, but dose received was not tracked for individuals. Out of those who participated in our survey, only 34 individuals (26%) in the intervention counties reported they had received health information more than once, and the most common subjects they had received information on were the Swedish health care system and self-care for common illnesses, for example colds. Reported contextual factors which may have influenced the reach and fulfillment of the IHA s’ goals could be the change in Swedish establishment policy in 2008, where health information for recently settled migrants was downplayed in favor of civic information. This resulted in a re-organization of how resettlement activities are planned and of actors responsible for implementing them.

### Limitations

The results of this study should be regarded in the light of its limitations.

*Selection bias* is a possible source of error, as the follow-up response rate was only 68%. At baseline, we found no systematic differences in participation between the genders or between age groups [13]. We assessed the dropouts at follow-up and found that more men than women, more younger than older individuals, and more individuals with low educational level than individuals with high educational level dropped out in the non-intervention group. The composition of the intervention group remained more stable. Studies have suggested that high dropout rates do not necessarily affect outcomes in any significant direction [48], but the risk cannot be completely ruled out. Research findings also show that socioeconomically disadvantaged persons are more prone to dropout from studies compared to those with a higher socioeconomic status.

*Misclassification* is another potential bias in this study. A non-differential misclassification of both exposed and unexposed individuals could have been the result of high mobility of the group and time lag in the register. In this case, the results may have been underestimated. However, we reduced this risk by omitting those who moved between the exposed and unexposed areas during the time period of the study, from the analysis.

*Confounding* could have flawed the findings of this study. However, some of this risk should have been controlled for in analyses adjusted for common social determinants (Tables 3 and 4).

## Conclusions

The main contribution of this article is the finding that an intervention based on provision of health information increased self-reported level of knowledge of healthy diet and physical exercise during the 2-year follow-up period. However, no statistically significant improvements in health status associated with the intervention could be measured in this study. This could be explained by limitations in the study, limitations in the theoretical assumptions underlying the intervention, and limitations in the implementation of the intervention. Poor health status remains a pertinent issue 2 years after refugee resettlement, and should be addressed more systematically. Further research is needed in order to understand health promoting factors better among recently settled migrants.

## Appendix 1

**Table 5:** Variable definitions/items in the questionnaire

Study variable	Questionnaire item
Unmet health care needs	Have you ever, during the last 3 months, regarded yourself in need of medical care, but not sought a doctor? Yes, several times
Poor mental health	General Health Questionnaire, 12 items 3 points or more
Poor dental health	How would you rate your current dental health? Quite poor/Very poor
Poor self-rated health	How would you rate your current general health condition? Quite poor/Very poor
Long-term illness	Do you have any lingering illness, difficulties following an accident, disability or other frailty? Yes
Low social participation	How often do you attend a meeting or another activity in any organization or group (for example sports organization, interest organization, mosque or church, women’s or men’s group)? Quarterly/More seldom or never
Low social participation	Take a stand on the following statements: a) Most people would use you if they got the chance b) Most people essentially try to be fair c) You can trust most people and d) You cannot be careful enough when you deal with other people Highest quartile of the distribution
Not received information on healthy diet/physical exercise/dental health	Have you received information on healthy diet/physical exercise/dental health? Yes, a little/No
Sex	Swedish population register
Age	Swedish population register
Low educational level	What education do you have? 7–9 years/1–6 years/None

## Abbreviations

CI: Confidence interval
GHQ: General Health Questionnaire
IHA: International Health Advisors
OR: odds ratio
SFI: Swedish for immigrants
